# Supplementary material for: Histopathologic Response Is a Positive Predictor of Overall Survival in Patients Undergoing Neoadjuvant/Perioperative Chemotherapy for Locally Advanced Gastric or Gastroesophageal Junction Cancers—Analysis from a Large Single Center Cohort in Germany
Source: Cancers (Basel). 2020 Aug 11;12(8):2244. doi: 10.3390/cancers12082244 (PMC7465424; doi:10.3390/cancers12082244)
Supplement: Supplementary file 1 [file cancers-12-02244-s001.pdf]

Supplementary Material: Histopathologic Response is A Positive Predictor of Overall Survival in Patients Undergoing Neoadjuvant/Perioperative Chemotherapy for Locally Advanced Gastric or Gastroesophageal Junction Cancers—Analysis from A Large Single Center Cohort in Germany

Rebekka Schirren, Alexander Novotny, Helmut Friess and Daniel Reim

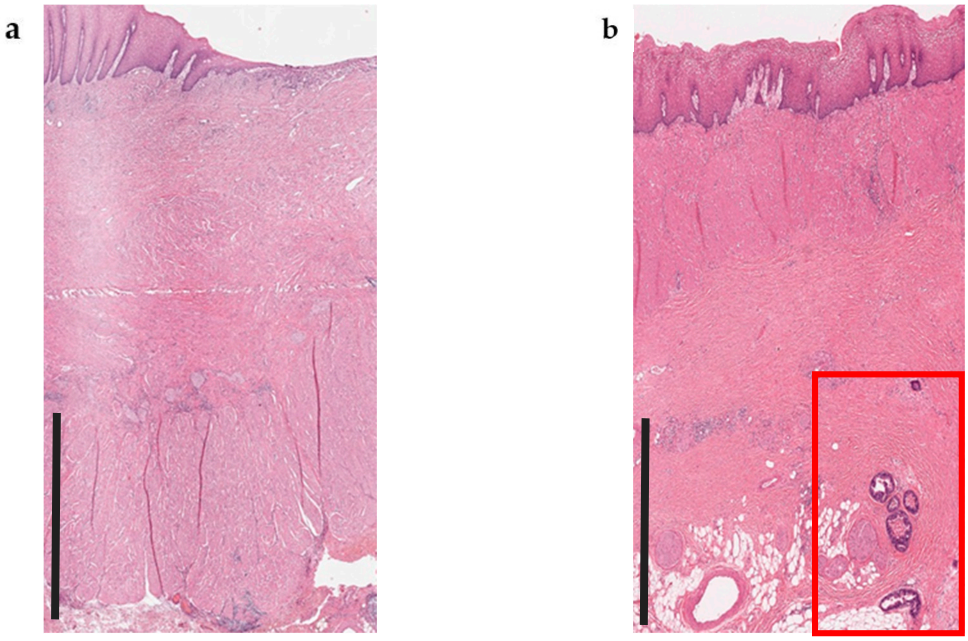

Figure S1. Histopathologic response grade Becker 1a and Becker 1b ((Sub-)total response (0–10% residual tumor cells in relation to tumor bed)). (**a**) In Becker 1a no residual tumor cells may be detected. (**b**) In Becker 1b less than 10% residual tumor cells are found (red inset-frame on the right picture). Scale bar: 1000 µm.

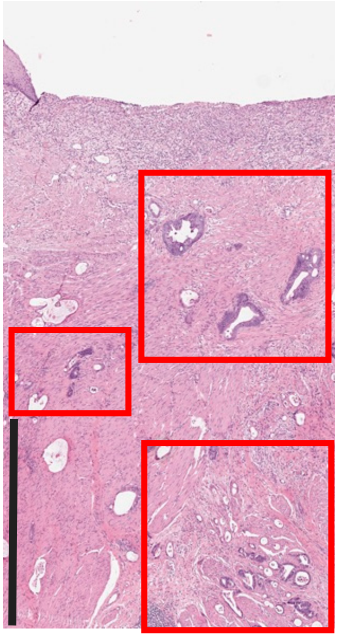

Figure S2. Histopathologic response grade Becker 2 (Partial response (10–50% residual tumor cells in relation to tumor bed)). Scattered viable tumor deposits are detectable throughout the tumor bed, which do not exceed 50% of the complete tumor area. Scale bar: 1000 µm.

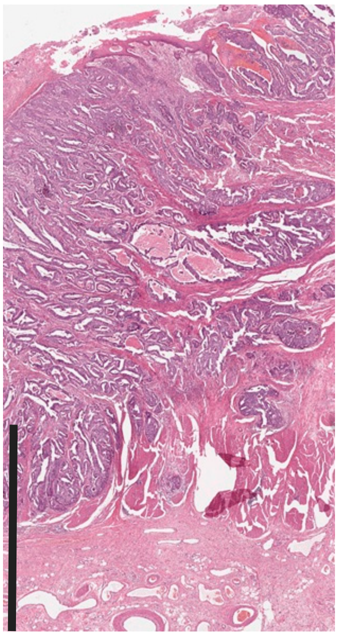

Figure S3. Histopathologic response grade Becker 3 (Non-response (>50% residual tumor cells in relation to tumor bed)). The tumor bed shows only some regression signs, most of the tumor consists of viable tumor cells. Scale bar: 1000 µm.

© 2020 by the authors. Licensee MDPI, Basel, Switzerland. This article is an open access article distributed under the terms and conditions of the Creative Commons Attribution (CC BY) license (http://creativecommons.org/licenses/by/4.0/).
